# Kinase Inhibition as Treatment for Acute and Chronic Graft-*Versus*-Host Disease

**DOI:** 10.3389/fimmu.2021.760199

**Published:** 2021-11-17

**Authors:** Lukas M. Braun, Robert Zeiser

**Affiliations:** ^1^ Department of Medicine I, Medical Center – University of Freiburg, Faculty of Medicine, University of Freiburg, Freiburg, Germany; ^2^ Faculty of Biology, University of Freiburg, Freiburg, Germany; ^3^ German Cancer Consortium (DKTK) Partner Site Freiburg, German Cancer Research Center (DKFZ), Heidelberg, Germany; ^4^ Comprehensive Cancer Center Freiburg (CCCF), University of Freiburg, Freiburg, Germany; ^5^ Centre for Biological Signalling Studies (BIOSS) and Centre for Integrative Biological Signalling Studies (CIBSS), University of Freiburg, Freiburg, Germany

**Keywords:** GvHD, stem cell transplant (SCT), kinases, ruxolitinib, JAK1 and JAK2 inhibitors, BTK - Bruton’s tyrosine kinase, ROCK

## Abstract

Allogeneic hematopoietic stem cell transplantation (allo-HCT) is a potentially curative therapy for patients suffering from hematological malignancies *via* the donor immune system driven graft-*versus*-leukemia effect. However, the therapy is mainly limited by severe acute and chronic graft-*versus*-host disease (GvHD), both being life-threatening complications after allo-HCT. GvHD develops when donor T cells do not only recognize remaining tumor cells as foreign, but also the recipient’s tissue, leading to a severe inflammatory disease. Typical GvHD target organs include the skin, liver and intestinal tract. Currently all approved strategies for GvHD treatment are immunosuppressive therapies, with the first-line therapy being glucocorticoids. However, therapeutic options for glucocorticoid-refractory patients are still limited. Novel therapeutic approaches, which reduce GvHD severity while preserving GvL activity, are urgently needed. Targeting kinase activity with small molecule inhibitors has shown promising results in preclinical animal models and clinical trials. Well-studied kinase targets in GvHD include Rho-associated coiled-coil-containing kinase 2 (ROCK2), spleen tyrosine kinase (SYK), Bruton’s tyrosine kinase (BTK) and interleukin-2-inducible T-cell kinase (ITK) to control B- and T-cell activation in acute and chronic GvHD. Janus Kinase 1 (JAK1) and 2 (JAK2) are among the most intensively studied kinases in GvHD due to their importance in cytokine production and inflammatory cell activation and migration. Here, we discuss the role of kinase inhibition as novel treatment strategies for acute and chronic GvHD after allo-HCT.

## Introduction

Patients suffering from hematological malignancies have only access to a very limited number of therapeutic interventions. Allogeneic hematopoietic stem cell transplantation (allo-HCT) is a potentially curative therapy for patients with hematological disorders (1, 2). Patients are pre-conditioned with chemotherapy or total-body irradiation to eradicate the underlying disease, followed by transplantation of donor stem cells. The allogeneic cells elicit an anti-malignancy immune reaction (3). However, besides the beneficial anti-tumor immune response, a major limitation of allo-HCT is acute and chronic graft-*versus*-host-disease (aGvHD, cGvHD) (4–7). GvHD is a life-threatening complication of allo-HCT and establishes if the transplanted cells recognize the host’s tissue as foreign. Host antigen-presenting cells (APCs) are activated and stimulate the donor cells, thereby causing cytokine release, strong immune cell activation and severe tissue damage (4, 5, 8–10). Acute GvHD is mainly based on T-cell activation and cytokine release, whereas B-cells are major players in cGvHD, which has features of autoimmune diseases and is often accompanied by organ fibrosis (5, 11). Acute and chronic GvHD are different diseases but share some similarities as both are inflammatory diseases initiated by APCs, followed by activation of alloreactive T- and B-cells, inflammation, tissue damage and organ failure (12). Acute GvHD is a risk factor for cGvHD development of (4, 11). Standard medications mainly rely on broad immunosuppression, which can on the one hand reduce GvHD activity, but on the other hand impair anti-malignancy immunity. There is an unmet need for specific and selective therapeutic strategies to control GvHD without disturbing beneficial immune responses after HCT. Kinase-mediated signaling pathways are among the most important signaling cascades to drive cytokine production and immune cell activation, thereby enhancing GvHD severity (13, 14). Many kinases share similarities, as e.g. JAK1/2, TAK1 and MAPK signaling are crucial for inflammatory cytokine signaling (15–17). TCR and BCR signaling, leading to cell survival, proliferation, migration and effector cytokine production, are mainly regulated by BTK and ITK, ROCK2, PI3K, mTOR, Syk and MEK (18–28). ROCK2, JAK1 and JAK2, as well as Syk, play a role in T-cell differentiation, including the induction of regulatory T-cells (27, 29–32). However, there is also a kinase involved in GvHD pathophysiology with a unique function, as ITPKB plays a pivotal role in regulating intracellular Ca^2+^ levels and T-cell survival (33–35). Based on the various functions of kinases in GvHD pathophysiology, it was concluded that tyrosine kinase inhibitors (TKIs) could be a promising strategy to control B- and T-cell activation and GvHD after allo-HCT (36). In general, TKIs block substrate phosphorylation, thereby limiting cellular downstream effects and pathways. These signaling cascades also include effector functions, e.g. the production of pro-inflammatory cytokines by T-cells (36). Since GvHD is mainly characterized by increased pro-inflammatory cytokines, systemic sclerosis and organ damage, inhibition of activated TK signaling could be a promising strategy to reduce disease severity and progression (36–38). Many small molecules were evaluated or are currently investigated for the treatment of GvHD, with some compounds now being applied as standard therapy. **Table 1** summarizes clinical trials about kinase inhibitors in GvHD, which are cited in this article. In this review, we would like to focus on kinases as novel and known targets in acute and chronic GvHD.

**Table 1:** Selected clinical trials of kinase inhibition in GvHD.

Trial number*	Treatment and kinase target	Trial description	Status, Outcome Measures, Comments
NCT02953678	Ruxolitinib;	REACH-1;	Completed;
	JAK1/2	Ruxolitinib combined with steroids for SR aGvHD;	ORR at day 28; CR, VGPR, PR; 6-month/3-month DOR; RR; FFS; relapse-related mortality; incidence/severity of AEs;
		Phase 2	71 participants; Single-cohort study
NCT02913261	Ruxolitinib;	REACH2;	Completed;
	JAK1/2	Safety/efficacy of Ruxolitinib vs. BAT in SR aGvHD;	ORR at day 28 and durable ORR at day 56; DOR; OS; cumulative steroid dose; event-free survival; FFS; NRM; MR; cGvHD incidence; PK; PROs;
		BAT selected by investigator;	310 participants; randomized open-label multi-center study
		Phase 3	
NCT03112603	Ruxolitinib;	REACH3;	Active;
	JAK1/2	Ruxolitinib vs. BAT in SR aGvHD after allo-HCT;	ORR of ruxolitinib vs. BAT in moderate to severe SR-cGvHD; FFS; change in modified Lee cGvHD symptom score; DOR; NRM; reduction in daily corticosteroid dose; MR; AEs; PK;
		Phase 3	330 participants; randomized open-label multi-center study
NCT02614612	Itacitinib; JAK1	Itacitinib in combination with corticosteroids in aGvHD; Phase 1	Completed; ORR; Itacitinib plasma concentrations; PK; 31 participants; open label study
NCT03320642	Itacitinib;	GRAVITAS-119;	Terminated by sponsor;
	JAK1	Itacitinib with calcineurin inhibitor-based intervention of GvHD prophylaxis;	Hematologic recovery; RFS; transplant-related mortality; immune reconstitution/engraftment; donor chimerism; OS; infections; 84 participants recruited; single group assignment
		Phase 1	
NCT03584516	Itacitinib;	GRAVITAS-309;	Recruiting;
	JAK1	Itacitinib and corticosteroids as initial treatment for cGvHD;	DLT; RR; Itacitinib plasma concentrations; time to response; OS; NRM; AEs; 431 participants; randomized, crossover assignment; ion part2, patients from placebo van cross over to experimental group after completion of primary analysis
		Phase 2/3	
NCT03846479	Itacitinib;	Itacitinib monotherapy	Active;
	JAK1	for low risk GvHD;	Minnesota standard risk clinical criteria; Ann Arbor Score 1; AEs; infectious complications; ORR;
		Phase 2	70 participants; single group assignment
NCT04070781	Itacitinib; JAK1 (plus Tocilizumab, IL6R)	Itacitinib and Tocilizumab for SR-aGvHD; Phase 1	Recruiting; MTD of Tocilizumab given with Itacitinib; Safety and tolerability; ORR; time to response; DOR; Infections; PFS; OS; steroid discontinuation; 24 participants; single group assignment; multi-center trial
NCT04446182	Itacitinib; JAK1 (with ECP)	Itacitinib and extracorporeal photopheresis for first-line therapy in cGvHD; Phase 2	Recruiting; Assess recommended phase 2 dose of Itacitinib with ECP combination; DLT; ORR; AEs; FFS; withdrawal of immunosuppressants; organ-specific response; GvHD severity; RR; OS; 58 participants; single group assignment
NCT04200365	Itacitinib;	Itacitinib for SR-cGvHD;	Recruiting;
	JAK1	Phase 2	Participants with SR-cGvHD after at least 6 months corticosteroids/other immunosuppressive therapies; combination therapies with Itacitinib; ORR; decrease or withdrawal of steroids; OS; AEs; quality of life; cGvHD progression/recurrence; RR;
			40 participants; Single group assignment; multi-center study
NCT02759731	Baricitinib; JAK1/2	Baricitinib in SR- cGvHD after allo-HCT; Phase 1; Phase 2	Recruiting; Safety, tolerability and efficacy of Baricitinib in patients refractory to steroids; 31 participants; non-randomized, open-label study
NCT04131738	Baricitinib; JAK1/2	Baricitinib for prophylaxis of GvHD; Phase 1	Recruiting; Cumulative incidence of graft failure; cumulative incidence of grade III-IV aGvHD; TRM; 26 participants; non-randomized, open-label study
NCT02195869	Ibrutinib; BTK	Safety and Efficacy of BTK ibrutinib in steroid dependent or refractory cGvHD; Phase 1b/2	Completed; Safety and tolerability (phase 1b/2b); ORR, CR, PR (phase 2); sustained response rate; corticosteroid requirement; improvement in Lee cGvHD symptom score; 45 participants; non-randomized multi-center open-label
NCT02959944	Ibrutinib;	iNTEGRATE;	Completed;
	BTK	ibrutinib/steroids vs placebo/steroids in new onset cGvHD; Phase 3	Response rate at 24 and 48 weeks; incidence of withdrawal of corticosteroids/all Immunosuppressants for GvHD treatment; improvement in Lee cGvHD symptom score; reduction of prednisolone dose; DOR; AEs;
			193 participants; randomized double blind multi-center study
NCT03474679	Ibrutinib; BTK	Ibrutinib in participants with steroid refractory/dependent cGvHD;	Active; ORR; CR; PR; sustained response; DOR; change of corticosteroid requirement; improvement of Lee cGvHD symptom score; AEs; clinical laboratory abnormalities; PK; metabolism; drug half-life;
		Phase 3	
			Single group assignment
NCT04294641	Ibrutinib; BTK	Front-line ibrutinib for newly diagnosed cGvHD;	Active/Recruiting; Efficacy of ibrutinib as first-line treatment for newly diagnosed cGvHD; ORR; safety; FFS; 24 month post-treatment survival;
		Phase 2	
			Pilot study; single group assignment
NCT02611063	Fostamatinib; SYK	Fostamatinib in cGvHD after allo-HCT;	Recruiting; MTD at day 60; TRM; incidence of cGvHD; relapse of cGvHD; B-cell activation; B-cell death; absolute B-cell numbers;
		Phase 1	
			Single group assignment
NCT02701634	Entospletinib; SYK	Entospletinib with systemic corticosteroids as first-line therapy in cGvHD;	Terminated; Best ORR; changes from baseline in Lee symptom scale (skin, mouth, eye, total); DOR; at least 50% reduction in systemic corticosteroid dose; FFS; AEs; study discontinuation; laboratory abnormalities;
		Phase 2	
			66 participants; randomized double-blind placebo-controlled study
NCT03640481	Belumosudil (KD025); ROCK2	Belumosudil in cGvHD after at least 2 prior lines of systemic therapy;	Recruiting; ORR; change in Lee symptom score; response in individual target organs; PR; CR; change in corticosteroid and calcineurin inhibitor dose; FFS; OS; activity change; cGvHD severity change; drug half-life; time to response; PK;
		Phase 2	
			Randomized multi-center open label study
NCT04930562	Belumosudil (KD025, BN101); ROCK2	Efficacy/Safety of Belumosudil in cGvHD; Phase 2	Recruiting; Individuals after at least first line of therapy; ORR; Single group assignment; open-label multicenter study
NCT02841995	Belumosudil (KD025); ROCK2	Safety, tolerability, activity of belumosudil in cGvHD; Phase 2	Active; ORR; PR; CR; AEs as measure of safety and tolerability; Dose-escalation open-label study
NCT00803010	Rapamycin; mTOR	GvHD prophylaxis after allo-HCT; Phase 2	Completed; Comparison of tacrolimus/rapamycin as novel GvHD prophylaxis vs tacrolimus/methotrexate; Incidence of aGvHD; incidence of increased Treg numbers; OS (2 years post-transplant); 74 participants; parallel assignment
NCT00928018	Sirolimus; mTOR	GvHD prophylaxis after reduced-intensity allo-HCT for lymphoma patients; Phase 3	Completed; Comparison of group 1 (tacrolimus, sirolimus, methotrexate), group 2 (tacrolimus, methotrexate) and group 3 (cyclosporine, mycophenolate mofetil) as GvHD prophylaxis regimen; OS; PFS; disease progression; non-relapse mortality; incidence of GvHD; 139 participants; parallel assignment; multicenter randomized trial
NCT01231412	Sirolimus; mTOR	GvHD prophylaxis after URD allo-HCT; Phase 3	Completed; GvHD prophylaxis with or without sirolimus after allo-HCT; grade II-IV aGvHD; incidence of extensive cGvHD; grade III-IV aGvHD; NRM; OS; relapse/progression rate; 174 participants; randomized study; parallel assignment
NCT02806947	Sirolimus; mTOR	BMT CTN 1501; Evaluation of steroid-free treatment of standard-risk aGvHD; Phase 2	Completed; Evaluation of sirolimus as alternative to prednisolone as up-front treatment for patients with standard-risk aGvHD; ORR; PR; CR; treatment failure; aGvHD; disease-free survival; OS; NRM; malignancy relapse; cGvHD, incautious complications; 127 participants; randomized multicenter open label study
NCT01106833	Sirolimus; mTOR	BMT CTN 0801; cGvHD treatment; Phase 2/3	Completed; Comparative study of sirolimus and prednisolone vs sirolimus and calcineurin-inhibitor and prednisolone; Proportion of treatment success; OS; PFS; FFS; relapse rate; rate of discontinuation of systemic immunosuppressive therapy; prednisolone dose; cGvHD severity; 151 participants; randomized open-label multicenter trial
NCT02891603	Pacritinib, Sirolimus; JAK2, mTOR	GvHD prevention combining pacritinib and sirolimus-based immunosuppression; Phase 1/2	Recruiting; Combination of pacritinib, sirolimus and tacrolimus to prevent serious GvHD; STAT activity in circulating CD4 T-cells; incidence of aGvHD; Single arm study
NCT00702689	Imatinib	Imatinib Mesylate in cGvHD with skin involvement; Phase 2	Completed; Change in range of motion (ROM); primary ROM response; AEs; ROM deficits; total skin score at baseline vs 6 months; cGvHD scores; lung function; change in immunosuppression; 20 participants; single group assignment
NCT01810718	Nilotinib	Safety and efficacy of Nilotinib in steroid refractory/dependent cGvHD; Phase 1/2	Completed; Phase 1: DLT; ORR; dose finding; phase 2: TTF; OS; biological evaluation (PDGF-R stimulating autoantibodies, fibroblast characteristics, changes of immune cell populations); 22 participants; prospective non-randomized open label multicenter study
NCT01155817	Nilotinib	Nilotinib in steroid dependent/refractory cGvHD; Phase 1	Completed; Determination of safety/tolerability in steroid refractory/dependent cGvHD; AEs; clinical efficacy in cGvHD; physical changes; daily corticosteroid requirement; treatment failure; cGvHD symptom burden; 33 participants; single group assignment

*All clinical trials are registered at https://clinicaltrials.gov; AEs, adverse events; aGvHD, acute GvHD; BAT, best available therapy; BTK, Bruton’s tyrosine kinase; cGvHD, chronic GvHD; CR, complete response; DLT, dose-limiting toxicities; FFS, failure-free survival; GvHD, Graft-versus-Host Disease; HCT, hematopoietic stem cell transplantation; JAK, Janus kinase; MR, malignancy relapse/progression; MTD, maximum tolerated dose; mTOR, mammalian target of rapamycin; NRM, non-relapse mortality; ORR, overall response rate; OS, overall survival; PFS, progression-free survival; PK, pharmacokinetics; PR, partial response; PROs, patient reported outcomes; ROCK2, rho-associated coiled-coil containing protein kinase 2; ROM, range of motion; RR, relapse rate; SR, steroid-refractory; SYK, Spleen tyrosine kinase; Tregs, regulatory T-cells; TRM, treatment-related mortality; TTF, treatment to failure time; URD, unrelated donor; VGPR, very good partial response.

## Janus Kinases 1 and 2 (JAK1/2)

One of the key players in mediating pro-inflammatory signaling is the Janus Kinase (JAK) 1/2. Signaling *via* JAK1/2 and signal transducer and activator of transcription (STAT) pathways are crucial for the stimulation of inflammatory cytokine production and the activation of a variety of immune cells during GvHD onset and progression (15).

**Figure 1:**
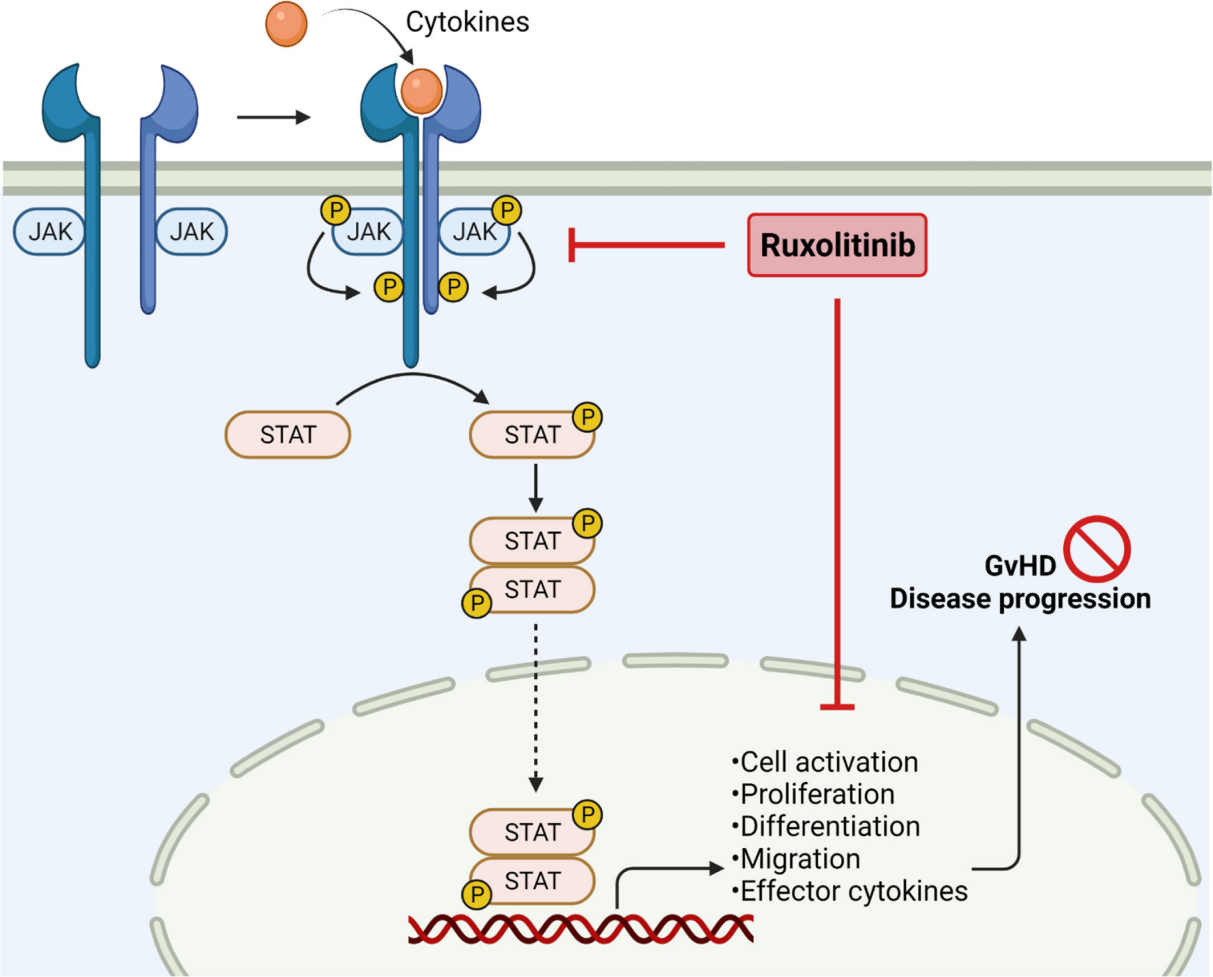
JAK2 inhibition in Graft-*versus*-Host Disease. Janus kinases are crucial to mediate extracellular signals. Binding of cytokines results in receptor dimerization and phosphorylation, subsequently phosphorylating STAT molecules by receptor tyrosine kinases. Phosphorylation of STAT leads to dimerization and translocation into the nucleus, followed by enhancing gene transcription. JAK/STAT signaling is important in regulating cell activation, proliferation, migration and effector cytokine production, thereby enhancing GvHD severity. JAK1/2 inhibition by ruxolitinib reduces pro-inflammatory signaling and cell migration, resulting in reduced GvHD disease progression. Created with Biorender.com.

**Acute GvHD:** The JAK/STAT signaling pathway has high importance in aGvHD onset and progression, as STAT1 and STAT3 are activated early after disease onset. The signaling plays a pivotal role in mediating T-cell activation and changes of the T-cell phenotype (39). Besides, JAK/STAT pathways are also important in the APC compartment in GvHD by influencing dendritic cell (DC) development, maturation, activation and migration into GvHD target organs (18, 40). JAK1 and JAK2 signaling can both be potently blocked with the selective inhibitor Ruxolitinib which has first been approved for the treatment of myelofibrosis by reducing pro-inflammatory signaling (41). Inhibition of JAK1/2 by Ruxolitinib does not only block DC activation and the common gamma chain downstream effects in T-cells (15), but also reduces the migration of neutrophil granulocytes into GvHD target organs (10, 42). Inhibition of JAK1/2 signaling by Ruxolitinib significantly reduced GvHD severity and increased the survival in a pre-clinical murine major-mismatch GvHD model (43). The findings could mainly be linked to a significant reduction of pro-inflammatory cytokine release *in vitro* and *in vivo*, reduction of donor T-cell infiltration into GvHD target organs and reduced allogeneic T-cell proliferation. The treatment also blocked allogeneic APC maturation and activation, thereby limiting T-cell proliferation. However, the blockade of JAK1/2 signaling also reduced T-cell proliferation stimulated by anti-CD3/CD28 activation beads. Direct effects on T-cells, including reduced activation and proliferation, could be linked to reduced STAT3 phosphorylation (**Figure 1**). Moreover, the number of regulatory T-cells (Tregs) was elevated in the intestine (43). Since Ruxolitinib application was seen to have cytopenia as side effects due to co-inhibition of JAK2, specific JAK1 inhibitors were designed to reduce cytokine signaling without side effects (44, 45). Itacitinib is a highly selective JAK1 inhibitor which has shown promising activity in inflammatory models, such as arthritis, inflammatory bowel disease and aGvHD (46). A preclinical study applied Itacitinib in a xenogeneic aGvHD humanized mouse model and found prolonged survival and reduced GvHD severity compared to control. Frequencies of CD4 and CD8 T-cells were lower on d21 and d28 after transplantation, whereas Treg frequencies increased. In a following GvL model, Itacitinib treatment reduced anti-leukemia immunity to some extent. However, more detailed analysis of JAK1 inhibition on T-cells would be necessary, including deeper phenotyping and effector function analysis (47). Baricitinib is another promising JAK inhibitor, blocking JAK1 and JAK2, and could prevent GvHD in a preclinical model (48). The investigators showed that single inhibition of JAK1 or JAK2 was not as effective as the double blockade with Ruxolitinib, hypothesizing that balanced blockade of both kinases is needed to optimally control GvHD. IFNγR and IL6R signaling, which is mainly regulated through JAK1/2, can be efficiently blocked with Baricitinib. Application of Baricitinib in murine GvHD resulted in 100 % survival, reduced early intestinal GvHD, and faster immune reconstitution with superior activity compared to Ruxolitinib. Mechanistically, Baricitinib treatment enhanced allogeneic Treg proliferation while blocking effector T-cell proliferation (48). GvHD-suppressive Tregs were increased by preservation of JAK3 signaling and increased STAT5 phosphorylation (49). Moreover, costimulatory molecule expression on allogeneic APCs was reduced. Most importantly, Baricitinib did preserve GvL activity and could control ongoing GvHD, making it a potential therapy for established GvHD and not only as prophylaxis treatment (48).

A first retrospective study included 95 patients receiving Ruxolitinib as a salvage therapy for steroid-refractory (SR-) GvHD and patients with severe intestinal or skin GvHD showed impressive response to JAK1/2 inhibition. Treatment with Ruxolitinib was liked to decreased pro-inflammatory serum cytokine levels and lower numbers of activated T-cells. The overall response rate (ORR) was 81.5 % and the overall survival (OS) of steroid-refractory aGvHD patients receiving Ruxolitinib was higher than ever reported for any other pharmacological therapy (50). This trial was followed by the REACH1 trial (NCT02953678), including patients with SR-aGvHD after HCT from any donor source. Comparable to the first results seen in patients, the time to response was 7 days and the ORR was 73.2 %. Also, the 6-month and 12-month OS was 51.0 % and 42.6 %, respectively, whereas the OS was lower in patients with grade III/IV aGvHD who received longer corticosteroid treatment before Ruxolitinib treatment. Mechanistically, biomarker analysis confirmed elevated hematopoiesis and a reduction of inflammatory cytokine release and signaling in patients receiving Ruxolitinib (51). The following REACH2 trial (NCT02913261) aimed to analyze the efficacy of Ruxolitinib in comparison to best available care in SR-aGvHD. Ruxolitinib significantly increased the median failure-free survival (FFS) and OS compared to control therapy, and the ORR at day 28 was significantly higher with Ruxolitinib treatment. The percentage of patients with complete response (CR) was at 34 % and 19 % under JAK1/2 inhibition or best available therapy, respectively. Comparable to side effects seen in the REACH1 trial, Ruxolitinib was again reported to cause thrombocytopenia (52). Besides JAK1 and 2 inhibition with Ruxolitinib, Itacitinib has shown promising preclinical efficacy in GvHD. Due to these findings and the hypothesis that selective JAK1 inhibition reduces side effects seen with Ruxolitinib, a phase I trial was initiated to determine if Itacitinib in combination with corticosteroids is safe and tolerable in patients with grade IIb-IVd aGvHD (NCT02614612). Treatment-naïve and SR aGvHD patients were included and distributed equally into two Itacitinib doses. Itacitinib was found safe to use; the most common nonhematologic AE was diarrhea, whereas hematologic AEs included anemia and thrombocytopenia. The d28 ORR was 75 % and 70.6 % of treatment naïve and SR aGvHD, respectively. Responses were seen across involved organs but median DOR was not reached in patients with treatment-naïve aGvHD. Upon Itacitinib treatment, corticosteroid doses could be reduced or discontinued in all patients. Overall, the study demonstrated that JAK1 inhibition with Itacitinib is effective and well tolerated in aGvHD. However, findings are limited due to small sample size and no comparator group (45). In another trial, Itacitinib was thought to be a promising prophylaxis treatment, as JAK/STAT blockade could limit T-cell migration into GvHD target organs; However, the study was terminated (NCT03320642). Itacitinib treatment is currently also investigated in more clinical trials as a therapy for low-risk GvHD (NCT03846479) or in combination with the anti-IL6R antibody Tocilizumab in aGvHD (NCT04070781).

**Chronic GvHD:** Besides application in aGvHD, Ruxolitinib treatment was also evaluated for the treatment of glucocorticoid-refractory cGvHD (53). Chronic GvHD occurs in 30-70 % of all patients undergoing allo-HCT and is treated with systemic glucocorticoids as first-line therapy (11, 54, 55). However, the disease becomes glucocorticoid-dependent or glucocorticoid-refractory in about 50 % of all patients, thereby significantly increasing the risk for poor outcomes (11, 53–55). Although the Bruton’s tyrosine kinase inhibitor ibrutinib is approved in the US and Canada as second-line therapy, responses are limited. Moreover, the efficacy of ibrutinib has not been demonstrated in a randomized clinical trial (11, 56, 57). In pre-clinical analysis, inhibition of JAK1/2 was shown to be an effective treatment not only in aGvHD, but also in cGvHD (50, 53). JAK1/2 is crucial for the initiation and progression of inflammation and cytokine signaling, both being major regulators of acute and chronic GvHD (15, 42, 43, 50, 58). Based on the positive results of Ruxolitinib in aGvHD in the REACH1 and REACH2 trial, it was evaluated in the REACH3 trial (NCT03112603) for the treatment of glucocorticoid-refractory or -dependent cGvHD in comparison to best available therapy (BAT, control) (51–53). The REACH3 trial is a phase III randomized open-label multi-center study of Ruxolitinib in comparison to ten other therapeutic agents. At the primary study end point, the OR was higher with Ruxolitinib (49.7 %) compared to control (25.6 %). A higher OR was observed with Ruxolitinib than any other control treatment in most organs, except for lung and liver cGvHD where responses were similar. Moreover, patients receiving Ruxolitinib had a significantly longer FFS than the control group (>18.6 months vs. 5.7 months). Also, the response on the modified Lee Symptom Scale was higher with Ruxolitinib (24.2 %) at 24 weeks compared to BAT (11.0 %). The investigators reported decreased dose of glucocorticoids in both groups over time, whereas the decrease was slightly greater in the Ruxolitinib group. Overall, the DOR was higher in the Ruxolitinib group compared to control treatment. Regarding the safety profile, adverse events (AEs) of any grade were slightly more often seen in the Ruxolitinib group compared to control, whereas adverse events of grade 3 and 4 were comparable in both groups (57.0 % vs. 57.6 %). Most commonly, Ruxolitinib treated patients experienced thrombocytopenia (15.2 %), anemia (12.7 %), neutropenia (8.5 %) and pneumonia (8.5 %). The safety profile was comparable to results seen in patients with aGvHD (53, 59, 60). Bacterial, fungal and viral infections were seen in both groups at a comparable incidence. In summary, the REACH3 trial showed that Ruxolitinib is superior over common second-line therapies for SR-cGvHD. Ruxolitinib was found being an effective treatment options for patients with moderate and severe SR-cGvHD (53). The selective JAK1 inhibitor Itacitinib is currently investigated as first-line therapy in cGvHD (NCT03584516), but results are not available yet. Since preclinical evaluation of Baricitinib in GvHD were promising, clinical trials were initiated to evaluate Baricitinib treatment in patients with cGvHD (NCT02759731) or as a prophylaxis treatment for GvHD after allo-HCT (NCT04131738). However, both trials are still recruiting and did not publish any results yet. Novel approaches combine JAK inhibitors with other therapies to enhance treatment efficacy. One trial investigates the combination of Itacitinib with corticosteroids or other immunosuppressive therapies in cGvHD (NCT04200365), or as combination with extracorporeal photopheresis (ECP) as first-line therapy in cGvHD (NCT04446182). In a comparable attempt, Ruxolitinib was combined with ECP in SR-cGvHD patients. Since both treatments alone did already show promising effects in GvHD, a combination therapy was thought to even elevate the therapeutic success. The 2-year survival rate was 75 % and the combination of both therapies was found safe to use with activity in at least a part of SR-cGvHD patients. However, this was only a single-center study and a detailed validation is needed in a prospective trial (61).

## Rho-Associated Coiled-Coil Containing Protein Kinase 2

**Figure 2:**
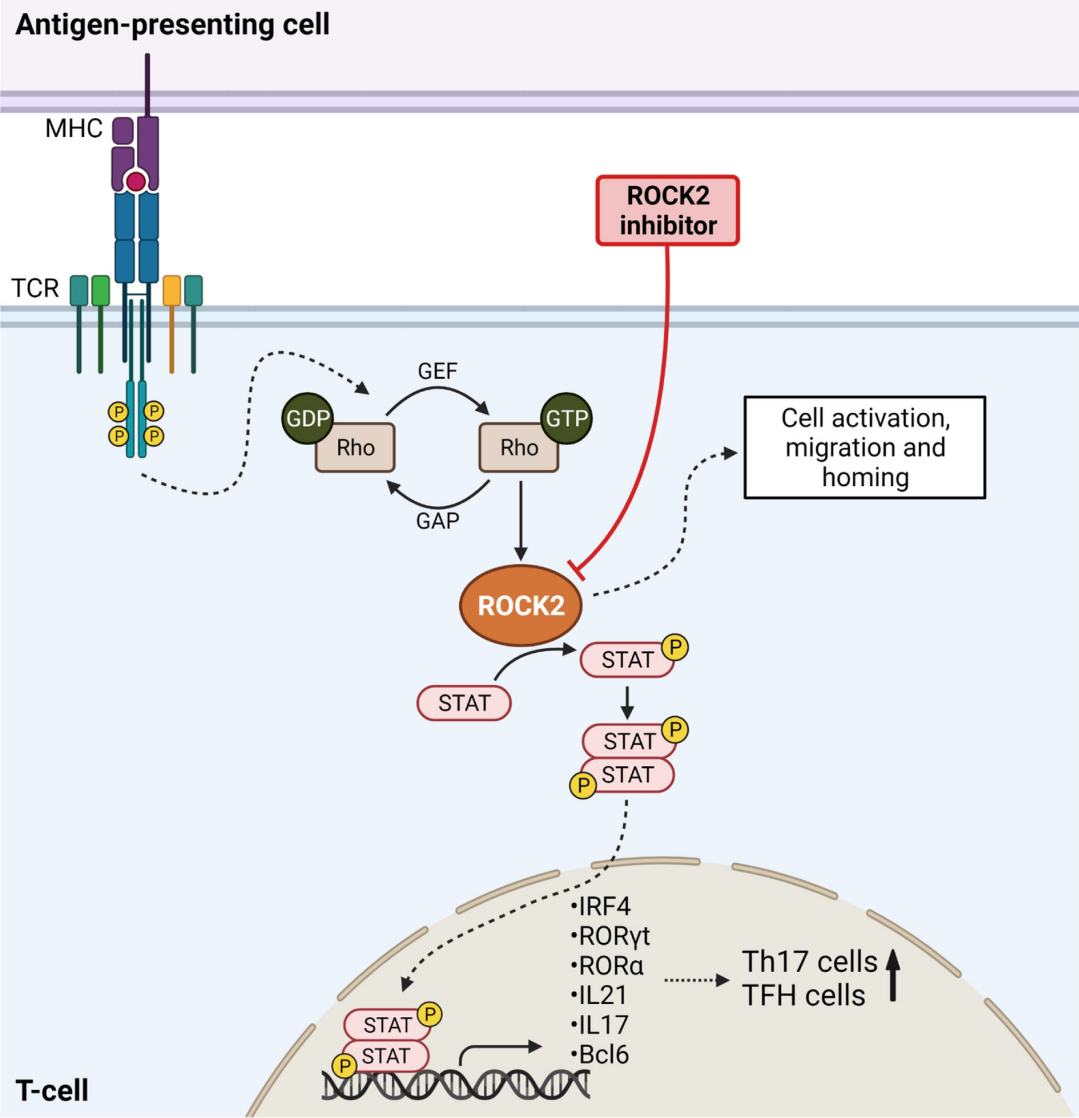
ROCK2 mediates Th17 differentiation in Graft-*versus*-Host Disease. TCR stimulation results in downstream ROCK2 activation, thereby phosphorylating STAT molecules. STAT is translocated into the nucleus to activate the transcription of Th17-specific transcription factors, thereby increasing the numbers of Th17 cells. ROCK2 activation further enhances the numbers of T follicular helper cells (TFH) and increases cell migration, activation and homing. Inhibition of ROCK2 blocks the differentiation of T-cells into TFH and Th17 cells and results in higher Treg numbers. Created with Biorender.com.

Signaling pathways mediated by Rho GTPase are important regulatory mechanisms of the T-cell mediated immune response, including TCR signaling and effector cytokine production (62). The rho-associated coiled-coil protein kinases 1 and 2 (ROCK1 and ROCK2) are serine-threonine kinases activated by Rho GTPases. Activation of ROCK1 and ROCK2 leads to phosphorylation of downstream molecules, including STAT3 and STAT5, to enhance the transcription of target genes (63). Target molecules regulated by ROCK2 include pro-inflammatory cytokines like IL-21 and IL-17 (23, 64). The ROCK2 signaling pathway has been shown to be important to regulate the balance between Th17 cells and Tregs. ROCK2 activation causes STAT3 phosphorylation, subsequently enhancing the expression of Th17-specific transcription factors, including interferon regulatory factor 4 (IRF4), RAR-related orphan receptor (ROR) γt and RORα (30–32, 65). Blockade of ROCK2 using its selective inhibitor belumosudil shifts the Th17/Treg balance towards regulatory T-cells through a STAT5-dependent mechanism (30, 32, 66). Regarding the potency of Tregs to reduce GvHD severity (67), ROCK2 was hypothesized being a promising target in GvHD (**Figure 2**) (30, 32).

**Chronic GvHD:** A preclinical study evaluated the effects of the ROCK2 inhibitor KD025 in cGvHD (66). Efficacy was first assessed in a bronchiolitis obliterans syndrome (BOS) model, in which the mice develop organ fibrosis associated with increased B-cell activation in the GC, also seen in cGvHD patients (66, 68). Upon treatment, the mice had improved resistance, elastance and compliance, together with decreased histopathology scores in the major GvHD target organs. However, cGvHD mice still had higher pathology scores than BM-only non-cGvHD animals. Also, collagen and immunoglobulin (Ig) deposition, both typically being increased in cGvHD multiorgan and BOS models (66, 68), were significantly decreased upon ROCK2 inhibition (66). Increased numbers of TFH cells and GC B-cells was previously been reported during murine cGvHD in the BOS model (68–71), all effects being reversed upon ROCK2 inhibition (66). Mechanistically, ROCK2 inhibition with KD025 significantly decreased STAT3 phosphorylation, whereas STAT5 phosphorylation was increased (66), both also reported in patients (32). Moreover, ROCK2 inhibition led to decreased expression of IRF4 and RORγt, crucial regulators for Th17 development (30–32, 65, 66, 72). Interestingly, the treatment did also reduce B-cell lymphoma 6 (Bcl6) expression, described as a TFH transcriptional regulator (66). The effects of KD025 in cGvHD were confirmed using a second minor mismatch Scl-cGvHD model. Skin pathology and GvHD scores were reduced together with a reduction of epidermal hyperplasia, infiltration of nucleated cells into the dermis and hair follicle destruction. Consistent with the BOS model, STAT3 phosphorylation and IRF4 levels were lower in the spleens of ROCK2 inhibitor treated mice (66). In order to translate the effects into the human system, peripheral blood mononuclear cells (PBMCs) were isolated from cGvHD patients and cultured in Th17-skewing culture medium either in the presence of KD025 or vehicle treatment. Comparable to cells from healthy individuals, ROCK2 inhibition reduced the production of IL-21, IL-17 and IFNγ in cells from cGvHD patients (32, 66). Important to note, targeted ROCK2 inhibition did not interfere with anti-leukemia immunity (66). The preclinical data suggested that ROCK2 inhibition could reduce cGvHD severity by both, downregulation of cytokine production and reduction of TFH cells, which are important for disease progression (66).

Based on promising preclinical data, the safety and efficacy of the selective ROCK2 inhibitor belumosudil was evaluated in clinical trials. The results of the ROCKstar study (NCT03640481) were published recently (73). The trial included patients after allo-HCT with persistent cGvHD manifestations indicating systemic therapy. Patient who received two to five lines of therapy (LOT) were included. The ROCKstar study evaluated belumosudil at 200 mg once (QD) or twice (BID) per day. The best ORR was 74 % (200 mg QD) and 77 % (200 mg BID), high responses were seen in all groups, and all affected organs demonstrated a response. A symptom reduction was seen in 59 % (QD) and 62 % (BID) of all patients. AEs were reported but not unexpected for cGvHD patients treated with immunosuppressive therapies. Overall, ROCK2 inhibition was found safe and well tolerated in patients suffering from cGvHD (73). A similar study is evaluating safety and efficacy of BN101 (belumosudil) in patients with cGvHD at a daily dose of 200 mg (NCT04930562). Results of this trial have not been published yet. Following the promising safety studies, a phase IIa trial was conducted as dose-finding study and to further analyze safety and efficacy of belumosudil in cGvHD patients previously treated with one to three prior LOT (NCT02841995) (30). The study included 54 patients in three different treatment cohorts, 200 mg daily, 200 mg twice per day and 400 mg daily. The ORR was comparable between all cohorts, ranging from 62 % to 69 %. Detailed organ analyses revealed complete remission (CR) in all affected organs, except for the lungs where a partial response (PR) was the best response. In general, the responses were achieved rapidly, with more than 75 % of the responses seen at eight weeks. Later organ responses were mainly seen in the lung and the eyes. The percentage of patients achieving FFS with response at 12 months was 24 % (30). The 12- and 24-month OS rate was 91 % and 82 %, respectively. Upon ROCK2 inhibition, 35 % of all patients experienced clinical improvement and 86 % could reduce or discontinue corticosteroids. Overall, belumosudil was well-tolerated and found safe to use. The main AEs were upper respiratory infections, diarrhea, fatigue, headache and hypertension. Belumosudil treatment was discontinued in three patients due to potentially drug-related AEs and four patients died during the study due to disease relapse and cGvHD progression; none of the deaths was related to the treatment. Mechanistically, blood analysis revealed increased Treg numbers together with decreased numbers of Th17 cells (30). In summary, the selective ROCK2 inhibitor belumosudil is a promising treatment option in cGvHD, targeting both, fibrosis and inflammation. The mechanistic results were comparable to the observations from the preclinical study (66). ROCK2 inhibition with belumosudil was granted Breakthrough Therapy Designation by the US Food and Drug Administration (FDA) and received FDA approval for the use in SR-cGvHD.

## Mammalian Target of Rapamycin (mTOR)

**Figure 3:**
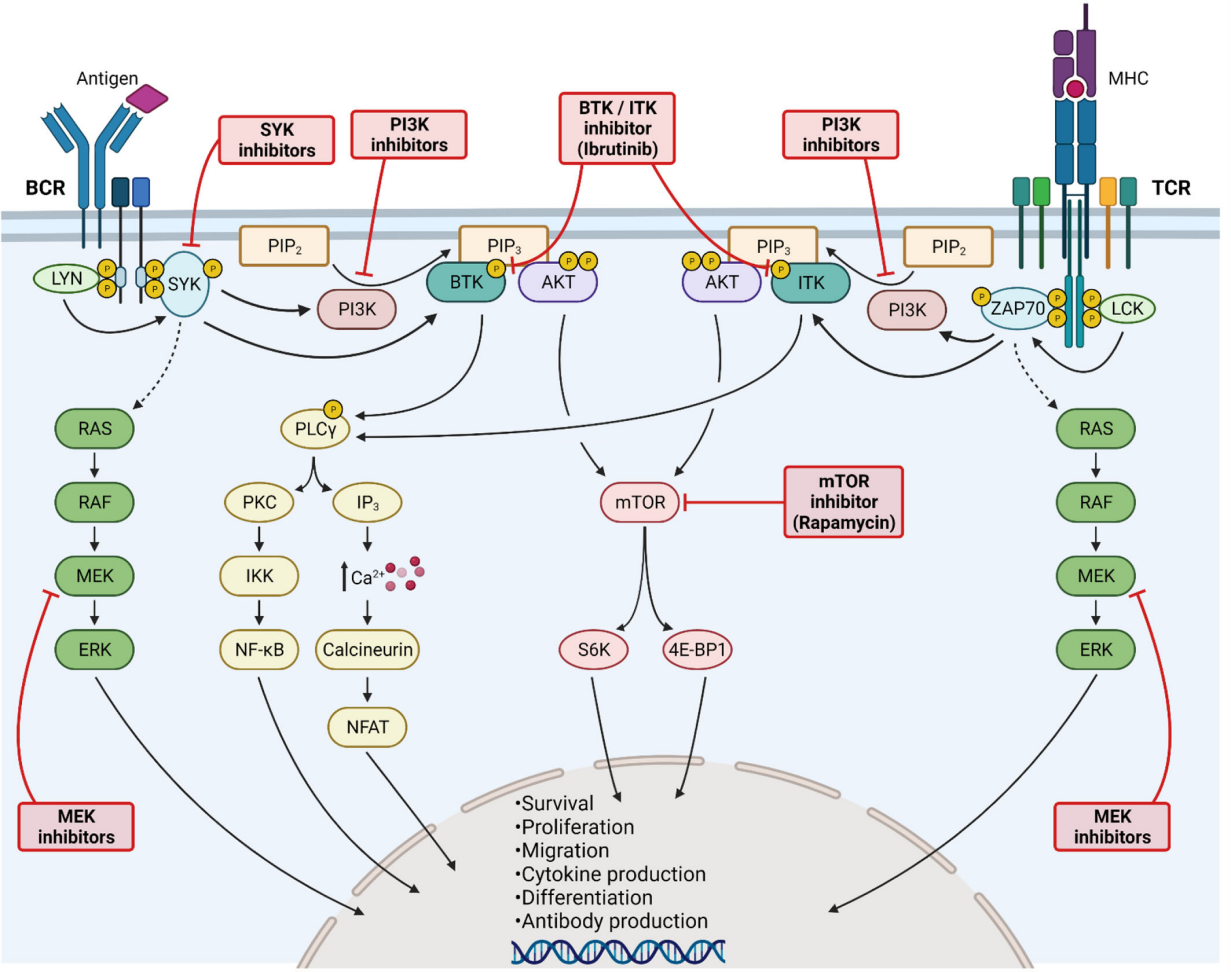
Kinase inhibition for Graft-*versus*-Host Disease treatment. Activation of B-cell (left side) and T-cell (right side) receptors leads to LYN/LCK phosphorylation, subsequently phosphorylating SYK and ZAP70. Both kinases further increase the downstream RAS/RAF/MEK/ERK signaling cascade. Activated SYK/ZAP70 stimulates also PI3K, as well as BTK and ITK. PI3K catalyzes the transformation of PIP_2_ into PIP_3_, being a binding site for BTK/ITK and AKT. BTK and ITK phosphorylate PLCγ, thereby enhancing PKC signaling and NF-κB translocation into the nucleus. Moreover, elevated calcium influx activates NFAT signaling. Activation of AKT stimulates mTOR signaling. All major kinase signaling pathways lead to cell survival, proliferation, differentiation and migration. Furthermore, cytokine and antibody production are enhanced. The signaling pathways can be blocked at various steps, including MEK, SYK, PI3K, BTK/ITK and mTOR inhibition. All kinase inhibitors have shown promising results in GvHD. Created with Biorender.com.

Donor T-cell activation and inflammatory cytokine secretion is a hallmark of GvHD after allo-HCT. Activation and effector functions of T-cells are tightly connected to the phosphatidylinositol 3-kinase/AKT/mammalian target of rapamycin (PI3K/AKT/mTOR) signaling cascade, which is crucial for the regulation of T-cell survival, proliferation, cell cycle progression, differentiation and metabolism (**Figure 3**) (7, 24–26, 74, 75). The major regulator of mTOR is a serine protein kinase formed of the mTORC1 and mTORC2 complexes (76, 77). The p70 ribosomal S6 Kinase (S6) is located downstream of mTOR and is the main signal transducer to enhance gene transcription and protein synthesis (7, 22). It is known that mTOR signaling is enhanced in GvHD, as T-cells isolated from allo-HCT recipients showed enhanced expression of Raptor and Rictor, both parts of the mTOR complex, and elevated S6 phosphorylation (78, 79).

**Acute GvHD:** In a preclinical study, transplantation of *Mtor*-deficient donor T-cells reduced aGvHD severity in mice, whereas more detailed analysis with *Raptor*-deficient allogeneic T-cells revealed that T-cell-mediated pathogenesis is dependent on mTORC1 but not on mTORC2 (79). Treatment with the mTORC1 inhibitor rapamycin (sirolimus) reduced GvHD severity in mice through reduction of pro-inflammatory cytokines and a blockade of T-cell proliferation and APC activity (79–81). Previous preclinical analysis revealed that rapamycin treatment is more effective in reducing murine GvHD mediated by CD8^+^ or TCR γδ^+^ T-cells than by CD4^+^ T-cells. Proliferation of CD8^+^ T-cells and production of Th1 and cytotoxic T-cell cytokines was inhibited, whereas Th2 cell differentiation was mainly unaffected. More detailed analysis was important for stratification of responsive patients, treatment time points and treatment combinations, as rapamycin did also reduce the GvL effect (80). Application of the immunosuppressant therapy cyclosporine A was shown to interfere with Treg activity, which is important to control GvHD. It was therefore crucial to clarify the effects of rapamycin on the different T-cell subsets, including Tregs. Preclinical and clinical studies revealed that rapamycin does not affect the expansion of Tregs (82–86). Treatment of mice suffering from aGvHD with rapamycin did not result in a survival benefit, however rapamycin enhanced survival of the mice when conventional T-cells (Tconv) and Tregs were transplanted together. The findings were confirmed by histopathological scoring. Rapamycin treatment and Treg transplantation reduced the proliferation of Tconv after allo-HCT. Mechanistically, rapamycin inhibited the proliferation of both, Tregs and Tconv, however Tregs were affected to a lesser extent and their immunosuppressive phenotype and FoxP3 expression was maintained. *In vivo* imaging confirmed that Treg proliferation is only slightly affected by rapamycin. In order to understand the differential impact of rapamycin on CD4^+^ T-cells, downstream analysis of mTOR was conducted by S6 and 4-EBP1 phosphorylation analysis after IL-2 stimulation and revealed minimal usage of mTOR signaling by Tregs. The findings of this study help to better understand the synergistic activity of Tregs and rapamycin in protection from aGvHD (87). It was also shown that rapamycin treatment preserves the thymic reconstitution of Tregs after allo-HCT, important to reduce GvHD severity (86). An early preclinical analysis hypothesized that PI3K blockade could be a promising strategy to reduce GvHD as *in vitro* treatment of donor lymphocytes with wortmannin reduced GvHD severity in mice (88). The role of the PI3K/AKT/mTOR pathway was further investigated in GvHD with an upstream blockade of PI3K using BKM120 or a novel dual PI3K/mTOR inhibitor BEZ235 (7). Both inhibitors blocked the signaling pathway as seen by decreased AKT and S6 phosphorylation. Both also reduced T-cell proliferation without affecting apoptosis, whereas the double inhibitor was more effective at lower concentrations. PI3K/mTOR pathway inhibition also reduced the secretion of Th1/Th2 effector cytokines, including IL-2, IL-6, IFNγ and TNFα. *In vitro* stimulation with anti-CD3/anti-CD28 increased early effector T-cells, which was reduced by BEZ235 treatment, but not by PI3K blockade alone. Based on promising *in vitro* data, major mismatch transplanted mice were treated with BEZ235; kinase inhibition significantly prolonged the survival of aGvHD mice and ameliorated GvHD (7). Castor et al. further investigated the role of PI3K in allo-HCT by transplanting PI3Kγ-deficient semi-allogeneic splenocytes in a murine GvHD model (89). Deficiency of PI3Kγ in the donor splenocyte compartment or inhibition of PI3Kγ with AS605240 significantly prolonged survival, increased body weight and reduced GvHD clinical scores in the recipient mice. Histological analysis revealed reduced damage of small intestine and liver and lower infiltration of CD11c^+^ and T-cells into the small intestine in the absence of PI3Kγ in donor splenocytes. PI3Kγ-deficiency was also associated with reduced pro-inflammatory cytokine secretion. Intravital microscopy showed decreased numbers of rolling and adherent cells in GvHD mice treated with AS605240 compared to vehicle. Together with hints for maintained anti-leukemia immunity, inhibition of PI3Kγ could be a novel strategy to suppress GvHD severity, although further analysis is necessary to better understand its role after allo-HCT (89). A recent preclinical study applied rapamycin in a GvHD model with 50 % MHC disparity and reported increased splenic leukocyte counts, including Tregs and myeloid-derived suppressor cells (MDSCs) (90). Whereas T-cell activation, exhaustion and cytokine secretion remained unchanged, rapamycin treatment rendered the MDSC population more immunosuppressive, reported the first time for mTOR blockade. MDSCs re-isolated from rapamycin treated GvHD mice had an increased immunosuppressive capacity towards alloantigen‐stimulated T-cells, confirmed by increased expression of iNOS, IDO and arginase-1. The importance of iNOS was underlined by application of a specific inhibitor, which abrogated the immunosuppressive phenotype of MDSCs. Since T-cell effector molecules remained unchanged with preserved GvL activity upon rapamycin treatment, the question how GvHD severity is reduced remains open. Nevertheless, the study described a novel and unknown role of mTOR kinase inhibition in GvHD on the myeloid compartment (90).

**Chronic GvHD:** The importance of mTOR signaling for GvHD pathogenesis is underlined by the finding of activating mTOR mutations in cGvHD patients which drive clonal CD4^+^ T-cell expansion and cGvHD development (91). Consistent with these findings, Sugiyama et al. highlighted in preclinical cGvHD models that mTOR inhibition, in contrast to cyclosporine A, does not increase the liability to cGvHD development. The investigators could see changed cGvHD scores in the skin and salivary glands upon rapamycin application compared to control (92).

Based on promising preclinical findings, a pilot clinical trial investigated the safety and efficacy of sirolimus as second-line therapy for GvHD treatment after allo-HCT (93). In total, 12 of 21 patients responded to the treatment, however, side effects were significant. AEs included thrombocytopenia, neutropenia and hemolytic uremic syndrome. Sirolimus had activity in patients with SR-GvHD, but dose optimizations were proposed due to severe toxicities (93). A combination of rapamycin with tacrolimus and low-dose methotrexate in GvHD patients was found feasible. In comparison to historical high-risk populations, the investigators reported lower rates of GvHD (94, 95). Following, a phase II trial combined rapamycin with tacrolimus as GvHD prophylaxis treatment after allo-HCT (NCT00803010). The combination treatment was superior over the control group and prevented high-grade aGvHD and moderate-severe cGvHD while promoting Treg reconstitution (96). A combination of sirolimus with calcineurin inhibitor could prevent GvHD in lymphoma patients after allo-HCT (97). Combining sirolimus/tacrolimus/methotrexate in lymphoma patients after allo-HCT did not affect OS, PFS and cGvHD, however, the addition of sirolimus prevented grade II-IV aGvHD (NCT00928018) (98). Recently, the efficacy of sirolimus was tested upon addition to standard GvHD prophylaxis (NCT01231412). Addition of sirolimus reduced grade II-IV aGvHD incidence, increased OS, but did not affect cGvHD (99). Whereas before-mentioned trials investigated sirolimus as prophylaxis treatment, Pidala et al. evaluated sirolimus for GvHD treatment (100). Sirolimus was tested vs prednisolone as initial treatment of patients with standard-risk aGvHD (NCT02806947). Day 28 CR/PR was comparable between both groups, however, CR/PR were significantly higher with sirolimus if compared to low-dose prednisolone. OS, disease-free survival, relapse and non-relapse mortality were comparable between both groups. Sirolimus reduced grade 2-3 infections, steroid exposure, hyperglycemia and enhanced patient-reported quality of life. Since sirolimus achieved comparable outcome at day 28 as prednisolone and spared steroid exposure, a confirming phase III trial is needed to also examine its efficacy in SR-aGvHD patients (100). Besides aGvHD, sirolimus was tested in combination with prednisolone or prednisolone/CNI in cGvHD (NCT01106833). CR/PR at 6 months, FFS and OS were the same at 2 years. Carpenter et al. concluded that sirolimus/prednisolone is an alternative, as a double-therapy is easier to administer and better tolerated than a triple-therapy (101). A first-in-human phase I/II clinical trial combines the JAK2 inhibitor pacritinib (PAC) with sirolimus and low-dose tacrolimus (PAC/SIR/TAC), aiming to reduce T-cell co-stimulation *via* mTOR and IL6 (NCT02891603). The effect of pacritinib/sirolimus was tested in human MLRs and a xenogeneic GvHD model and consistently suppressed allogeneic T-cell proliferation and GvHD severity (102, 103). STAT3 and S6 phosphorylation were reduced upon treatment, confirming JAK2/mTOR inhibition. In mice, the treatment reduced Th1 and Th17 cells while increasing Tregs. Anti-leukemia and anti-CMV immunity were preserved. Following, the PAC/SIR/TAC combination will be tested in the ongoing phase II trial (102). Overall, targeting mTOR signaling with sirolimus and blocking the PI3K pathway are both promising and established strategies to reduce acute and chronic GvHD either as prophylaxis or treatment of an established disease and may be preferred to other regimens for patients after allo-HCT.

## Bruton’s Tyrosine Kinase (BTK) and Interleukin-2 Inducible T-Cell Kinase (ITK)

Dysregulation of T- and B-cell activation and proliferation, enhanced antibody production, inflammation and organ damage are typically seen during GvHD development (104, 105). Stimulation of BCR and TCR and the subsequent activation of downstream pathways is crucial for GvHD induction after allo-HCT (106, 107). Bruton’s tyrosine kinase (BTK) is part of the BCR signaling complex and kinase activation is necessary for survival, migration and proliferation of B-cells (19). Genetic BTK-deficiency results in a loss of peripheral B-cells and a blockade of immunoglobulin production (108). Activation of BTK subsequently phosphorylates phospholipase Cγ2 (PLCγ2), thereby facilitating further downstream effects like NF-κB and NFAT activation to enhance survival, proliferation and migration of B-cells (14, 19, 20). The interleukin-2 inducible T-cell kinase (ITK), another Tec family kinase, has functional similarities with BTK but is crucial for TCR signaling (20, 21). Comparable to BCR signaling, ITK is important for PLCγ2 activation downstream of the TCR, thereby facilitating signaling through NF-κB, NFAT and MAPK to activate T-cells, enhance proliferation and promote cytokine production (14, 20, 109, 110). ITK is important for driving the secretion of IL-2, IL-17 and Th2 cytokines, all being associated with cGvHD pathogenesis (11, 20, 111–114). Regarding the importance of T-cells and B-cells in both, acute and chronic GvHD, inhibition of BTK and ITK could be a promising strategy to inhibit GvHD development by blocking B- and T-cell activation, thereby hindering severe inflammation and fibrosis (14). Ibrutinib is an FDA-approved inhibitor, blocking both ITK and BTK, which was first approved for the use in lymphocytic leukemia (**Figure 3**) (21).

**Acute GvHD:** Since ibrutinib has inhibitory effects on both, BTK and ITK, a preclinical study determined the ability of ibrutinib to target donor-derived T-cells in an aGvHD model. For exclusion of donor B-cells, T-cell depleted BM was transplanted together with T-cells from B-cell KO donor mice. Treatment with ibrutinib improved aGvHD clinical scores and survival of mice, whereas the latter was not significantly changed. Although T-cell proliferation and activation was unaffected upon ibrutinib treatment, experiments with B-cell-deficient donor mice confirmed an effect of ibrutinib on donor T-cells after allo-HCT (14).

**Chronic GvHD:** Since application of ibrutinib led only to slight improvement in acute GvHD, ITK and BTK inhibition with ibrutinib was evaluated in a pre-clinical cGvHD model (104). Mice receiving the treatment survived significantly longer compared to vehicle, did not develop ascites and had delayed onset of proteinuria. Both was associated with cGvHD in mouse models. However, proteinuria was only prevented with longterm treatment. Notably, ibrutinib suppressed cGvHD development and prolonged survival if given after the disease was already established (14). Mechanistically, ibrutinib inhibited B-cell proliferation and co-stimulatory molecule expression, known to be crucial for GvHD pathogenesis (14, 115). T-cell proliferation was not affected. CD4^+^CD8^+^ thymocytes were increased, pointing towards enhanced immune reconstitution upon ibrutinib treatment. These findings were not confirmed by lineage staining after allo-HCT and need further detailed analysis to substantiate this hypothesis (14). In an additional scleroderma model (116) with prophylactic ibrutinib treatment prior to allo-HCT, the investigators found that inhibitor treated mice showed less cGvHD symptoms, reduced skin damage, less alopecia and lower GvHD scores after allo-HCT. Prophylactic effects were only seen with high-dose treatment. Ibrutinib treatment did also enhance the reconstitution of B-cells and reduced T follicular helper (TFH) cells after allo-HCT. Protective effects of ibrutinib were confirmed in an aGvHD model transitioning into cGvHD (14). In a second preclinical study, ibrutinib was applied in sclerodermatous cGvHD starting on day 25 after allo-HCT when the first symptoms became apparent (70, 117). The investigators found reduced clinical signs of cGvHD. These findings were accompanied by improved progression-free survival upon ibrutinib treatment. Moreover, ibrutinib application diminished B- and T-cell infiltration into lung and kidney and led to lower GvHD pathology scores in these cGvHD target organs. The investigators applied a second model, aiming to understand the effects of ibrutinib treatment on bronchiolitis obliterans (BO) in cGvHD (70). The treatment started on day 28 after allo-HCT and resulted in reduced pulmonary resistance and elastance, better compliance and lower lung fibrosis. Analysis from ibrutinib treated mice was comparable to non-GvHD mice. Withdrawal of therapy led to a loss of benefit, indicating that ibrutinib treatment need to be applied continuously. Contrary to the previously reported study, prophylactic ibrutinib treatment could not effectively combat cGvHD or BO (14, 70). To further clarify the role of BTK and ITK in cGvHD, the investigators transplanted WT bone marrow together with ITK-deficient T-cells into allogeneic recipients. Donor-derived T-cells are known to be important for cGvHD development. ITK deletion could reverse cGvHD signs in the lungs to values comparable with non-GvHD animals and ibrutinib treated cGvHD mice. Comparable results were seen when XID bone marrow, which lacks BTK, was used as allogeneic graft. B-cells driving cGvHD development rather arise from the transplanted bone marrow. These experiments highlight that both Tec kinases, ITK and BTK, play a role in cGvHD development. Analysis of *ex vivo* ibrutinib treated cGvHD patient-derived CD4^+^ T-cells revealed reduced activation upon kinase inhibition. Reduced activation was also seen in patient-derived B-cells with lower BTK, ERK1/2 and PLCγ2 phosphorylation (70). Comparing both described preclinical studies, all models found that ibrutinib treatment affected B-cell activation and differentiation, whereas the effects on T-cells were variable. Clinical GvHD scores were improved in all models and effective in both, prophylactic treatment and treatment of established disease (20).

The promising preclinical data paved the way to further investigate ibrutinib treatment in cGvHD. A phase Ib/II study was conducted to determine safety and efficacy of ibrutinib in patients who failed at least one LOT for cGvHD (NCT02195869) (20, 118). Ibrutinib did not show dose-limiting toxicities. At a median follow-up of 13.9 months, 29 % of patients were still receiving the drug, whereas 71 % of patients discontinued due to adverse events (AEs), cGvHD progression and patient decision. Most AEs were low grade and well manageable and led to dose reductions. A total of 29 patients (69 %) developed infectious complications of any grade. The ORR was 76 % and 71 % of responders showed a response for more than 20 weeks. Even responses were seen in all cGvHD target organs. Corticosteroid therapy could be reduced with ibrutinib treatment. Detailed mechanistic analysis showed strong inhibition of BTK and ITK, reduced pro-inflammatory mediators in the serum, less germinal center (GC) B-cells and total B-cells and reduced numbers of Th17 and TFH cells (20, 118). Promising data from this trial led to the iNTEGRATE phase III clinical trial investigating ibrutinib in combination with prednisone in patients with newly diagnosed moderate to severe cGvHD after allo-HCT (NCT02959944). Response rate was slightly higher in the ibrutinib group and corticosteroids could be withdrawn at 21 and 24 months in the ibrutinib arm. Patients receiving ibrutinib had improved Lee symptom scores. Another phase III trial evaluates the efficacy of ibrutinib in patients with SR-cGvHD (NCT03474679) and an additional phase II trial is currently recruiting to investigate ibrutinib as first-line therapy for newly diagnosed cGvHD who did not receive any systemic treatment for cGvHD (NCT04294641). So far, both of the last mentioned trials did not publish any results yet.

Based on very promising preclinical and clinical trial data, ITK and BTK inhibition with ibrutinib could be a very potent therapy in chronic GvHD. However, additional detailed analysis of the underlying mechanism is necessary to improve the therapeutic success. Since clinical trials focus on cGvHD, further analysis and preclinical models are needed to investigate the role of ITK blockade in aGvHD.

## Spleen Tyrosine Kinase (Syk)

The non-receptor cytoplasmic spleen tyrosine kinase (Syk) was hypothesized being an important regulator of GvHD as it has functions in transmitting signals from surface receptors, including Toll-like receptors (TLRs) (119), Fc receptors (120), as well as chemokine receptors (27, 121, 122). Moreover, Syk activation is known to be crucial for TCR signaling upon peptide binding, as well as playing an important role in T-cell lineage commitment, mainly for Th17 responses which are known to be involved in GvHD pathophysiology (27, 29, 123). Based on the knowledge that Syk inhibition, e.g. using Fostamatinib, has beneficial effects in inflammatory diseases, the relevance of Syk in GvHD was further evaluated (**Figure 3**) (27, 124–126).

**Acute GvHD:** In a preclinical murine aGvHD model, daily treatment with Fostamatinib led to significantly improved survival, reduced histopathology scores and reduced pro-inflammatory serum cytokine concentrations. Fostamatinib treatment did not interfere with donor lineage engraftment and immune reconstitution. Syk phosphorylation is rapidly increased upon CD3/CD28 stimulation of T-cells and higher pSyk 525/526 levels were also seen in T-cells isolated from aGvHD mice. Using luciferase transgenic T-cells, Syk inhibitor treatment was found to reduce alloreactive donor T-cell expansion *in vivo*. The findings were confirmed by CFSE staining, indicating reduced T-cell proliferation *in vivo* upon Syk inhibition. Besides blockade of T-cell proliferation, Syk inhibition also reduced T-cell migration towards CXCL12. Contrary to previous findings about the importance of Syk in T-cell lineage commitment, Fostamatinib treatment did not change the percentage of Th2 and Th17 cells after allo-HCT. Effects on T-cells could be further affected by APCs as Syk inhibition was also connected to diminished costimulatory molecule expression and reduced DC migration *in vivo* and *in vitro*. Although proliferation and effector cytokine secretion of allogeneic donor T-cells was significantly reduced by Fostamatinib, the GvL effect was preserved, as confirmed by *in vivo* bioluminescence imaging using luciferase transgenic leukemia cells and *ex vivo* killing assays. Overall, pharmacological inhibition of Syk was found being a novel treatment strategy in aGvHD by reducing T-cell expansion and costimulation while preserving anti-leukemia immunity (27).

**Chronic GvHD:** Besides its role in TCR signaling, Syk is also involved in BCR signaling and controlling cell migration and adhesion (36). Knowing the importance of B-cells in cGvHD, Syk inhibition was also hypothesized being a major regulator of cGvHD pathophysiology (8, 11, 36, 69, 127). In a cGvHD model with multiorgan involvement, Syk-mediated BCR signaling in allogeneic B-cells was validated being crucial for cGvHD development (69). The investigators isolated B-cells from cGvHD animals at day 60 after allo-HCT and showed increased Syk phosphorylation at Y348 (69). Comparable results were found in B-cells from cGvHD patients (107). The importance of Syk signaling was further evaluated using Syk-deficient allogeneic BM donors for a model of cGvHD with multiorgan involvement. The mice did not develop pulmonary dysfunctions after transplantation with Syk KO BM, whereas Syk-deficient T-cells did not attenuate cGvHD severity. Additionally, Syk was not only important in the initiation of cGvHD, but also in disease progression as pulmonary dysfunction was reversed upon tamoxifen-induced Syk depletion. The Syk inhibitor Fostamatinib (126, 128), was applied during active disease and reduced cGvHD severity in the lung and improved pulmonary dysfunctions (69). Contrary, improvement of skin inflammation was not seen in three Scl-cGvHD models upon Fostamatinib treatment. However, one model showed attenuated skin GvHD and clinical GvHD scores upon Syk inhibition (69). In an additional study of Scl-cGvHD, the investigators proved that Syk phosphorylation is increased in T- and B-cells, as well as in CD11b^+^ cells, after allo-HCT (129). Early treatment with Fostamatinib reduced the severity and fibrosis of Scl-cGvHD and the expression of pro-inflammatory molecules in the skin. Moreover, the migration of antigen-specific memory CD4^+^ T-cells and the proliferation and activation of allogeneic CD4^+^ and CD11b^+^ cells was suppressed, comparable to results seen in the aGvHD setting (27, 129). Since this data is contrary to a before-mentioned study, it is important to note that effects of Fostamatinib were mainly seen if the treatment was applied early after cGvHD induction (69, 129). When B-cells isolated from patients were treated with Fostamatinib *in vitro*, the drug preferentially killed cGvHD B-cells seen by increased apoptosis if compared to non-cGvHD control B-cells (69, 107). Additionally, Syk inhibition blocked the differentiation of CD4 T-cells into Th2 and Th17 phenotypes (8, 69, 129). This was different if compared to Syk inhibition in aGvHD described above (27). Using the second-generation highly selective Sky inhibitor entospletinib, Poe et al. demonstrated that inhibitor treatment blocked the development of eye GvHD and also significantly reduced hair loss in GvHD animals. Besides reducing GvHD severity, entospletinib treatment led to improved reconstitution of monocytes, B-cells and T-cells at 28 and 42 days after allo-HCT. Moreover, pathogenic activated GL7^+^ B-cells and Th2 cells were diminished upon Syk inhibition, both playing a role in acute and chronic GvHD (130–132). T-cell differentiation was changed upon entospletinib treatment with increased numbers of Tregs and a reduction of Th17 cells. Overall, the treatment significantly prolonged the survival of the mice after allo-HCT and reduced skin inflammation and GvHD severity (130).

Taken together, the data derived from genetic and pharmacological approaches in pre-clinical murine GvHD models clearly indicate that Syk plays an important role in GvHD pathophysiology. Pharmacological targeting of Syk could be a novel attempt to treat GvHD. Based on these promising findings of Syk inhibition with Fostamatinib *in vitro* and from preclinical *in vivo* models, the efficacy of Fostamatinib to prevent and treat cGvHD after allo-HCT is currently evaluated in a phase I trial (NCT02611063). Another phase II trial was investigating the efficacy and tolerability of entospletinib in combination with systemic corticosteroids cGvHD as first-line therapy, however, the study was terminated (NCT02701634). Based on pre-clinical studies, it would also be interesting to evaluate effects of Syk inhibition in patients with aGvHD. However, ongoing clinical trials first focused on cGvHD, which might be due to the importance of Syk in BCR downstream signaling, whereas it is not essential for TCR downstream events.

## Platelet-Derived Growth Factor Receptor

**Chronic GvHD:** cGvHD is often presented with dermal fibrosis and sclerosis, associated with the presence of stimulatory anti-platelet-derived growth factor receptor (PDGFR) antibodies, suggesting a direct link between skin fibrosis and PDGF signaling (133). PDGFR stimulation causes enhanced collagen production, which could contribute to organ damage (134). Besides PDGFR stimulation, TGFβ is known as an important mediator of fibrosis in cGvHD (135, 136) and inhibition of both reduced pro-fibrotic activity and pulmonary fibrosis in experimental models (137, 138). Imatinib was first developed as a treatment for BCR-ABL positive CML, but also inhibits PDGFR and was therefore hypothesized as a novel therapeutic intervention in cGvHD by reducing fibrosis (133, 139). In preclinical analyses of bleomycin-induced fibrosis, imatinib inhibited the development of dermal fibrosis by reducing COL1A1, COL1A2, and fibronectin 1 transcription. Moreover, the induction of extracellular matrix proteins, stimulated by PDGF and TGFβ, was reduced upon imatinib application (139, 140). Additionally, imatinib could also reduce fibrosis in kidney and liver, target organs of cGvHD (140). Based on this, Belle et al. applied imatinib in a murine model of Scl-cGvHD but found limited impact besides reduced PDGFR phosphorylation. T-cell proliferation was slightly inhibited, but GvHD scores were unchanged (141). Contrary to these findings, another study reported that both, imatinib and nilotinib prevent the development of Scl-GvHD in mice (142). Nilotinib is a second-generation TKI targeting BCR-ABL and PDGFR with a higher affinity than imatinib (36). Both TKIs inhibited dermal fibrosis and reduced dermal thickness if given as prophylaxis treatment. Additional to these findings, GvHD was also significantly reduced when imatinib or nilotinib was given after onset of clinical disease (142). Serum analysis of patients treated with nilotinib showed reduced inflammatory cytokine secretion, including TNFα, IFNγ and IL-2 (143). Using *ex vivo* cultures, GvHD-derived fibroblasts expressed higher levels of collagen genes, which was significantly reduced upon nilotinib application. Confirming *in vitro* data, skin analysis from cGvHD patients showed decrease of COL1α1 and COL1α2 protein levels, TGFβ inhibition and p-Smad2 reduction upon treatment with nilotinib (144). Both, imatinib and nilotinib showed efficacy in clinical trials. Treatment of SR-cGvHD with skin involvement led to improved joint range of motion and better skin scores (NCT00702689), proposing imatinib as possible salvage therapy for SR-cGvHD (133). Olivieri et al. reported high OR of imatinib treatment in patients with refractory cGvHD who previously failed at least two LOTs (145). Comparable, scleroderma symptoms disappeared upon imatinib application and the treatment was well-tolerated (146). A retrospective study confirmed a beneficial activity of imatinib as a salvage therapy in Scl-cGvHD (147). After introduction of the second-generation TKI nilotinib, the compound was tested in SR-cGvHD (NCT01810718). The 2-year OS was 75 % with FFS of 30 %. Based on promising long-term outcomes and well-manageable side effects, nilotinib was hypothesized as a promising treatment in SR-cGvHD (148). Another trial investigating safety and efficacy of nilotinib in SR-cGvHD did not post any results yet (NCT01155817). Taken together, pre-clinical and clinical data confirm the TKIs imatinib and nilotinib as promising therapeutic interventions for cGvHD with organ fibrosis.

## Inositol 1,4,5-Triphosphate 3-Kinase B (ITPKB)

**Figure 4:**
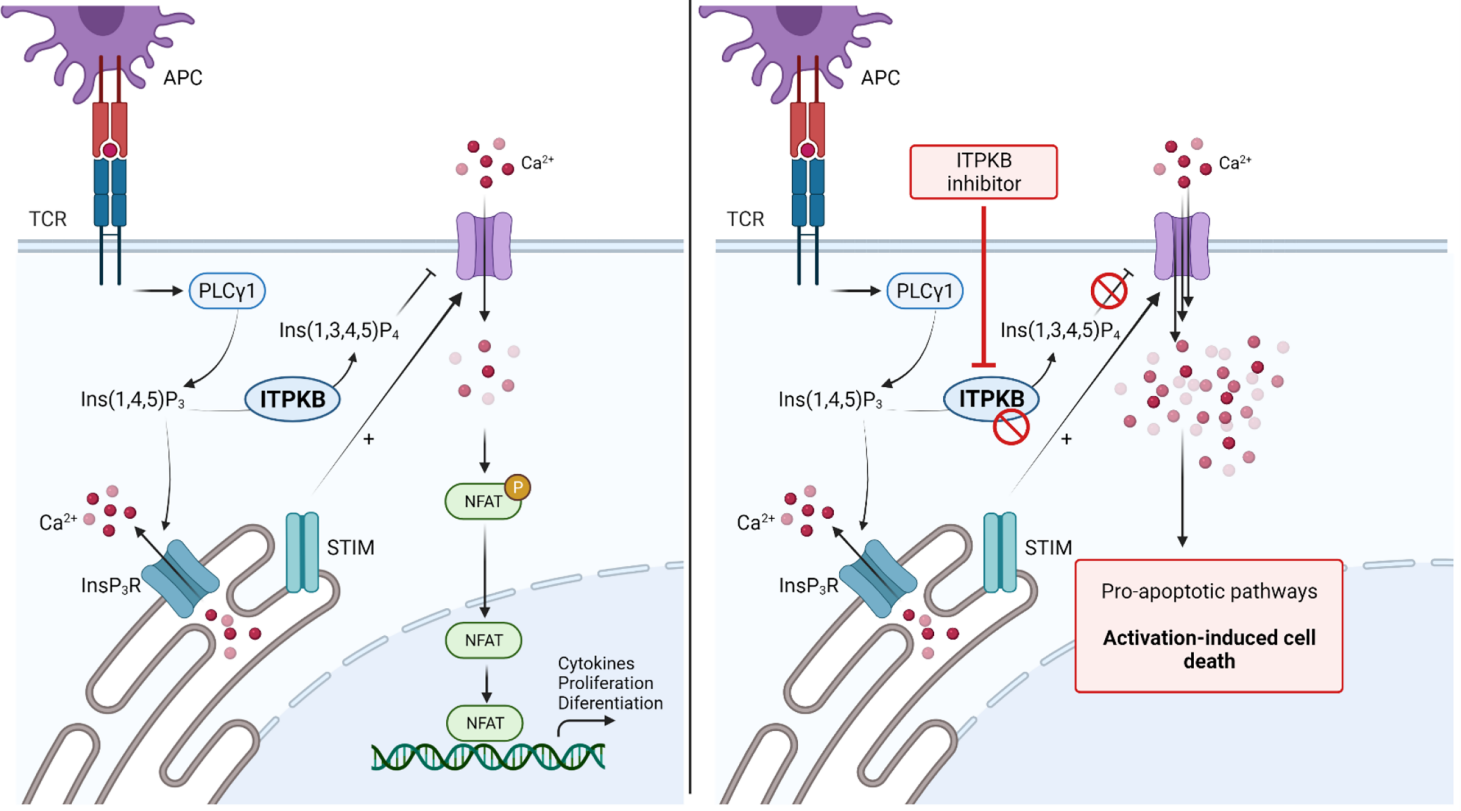
Blockade of Inositol 1,4,5-triphosphate 3-kinase B (ITPKB) as a novel treatment for Graft-*versus*-Host Disease. TCR stimulation activates downstream PLCγ, thereby increasing intracellular IP_3_ levels. Binding of IP_3_ to IP_3_R activates the release of Ca^2+^ from intracellular storage compartments. Furthermore, STIM stimulate the influx of extracellular Ca^2+^ to activate NFAT signaling and gene transcription. ITPKB is a rate-limiting step as it catalyzes the formation of IP_4_ from IP_3_. IP_4_ acts as a control mechanism for calcium signaling by blocking the respective channels in the extracellular membrane. Genetic deletion or inhibition of ITPKB disturbs this control mechanism and leads to increased calcium influx, which stimulates pro-apoptotic signaling pathways, leading to activation-induced cell death. Since ITPKB is predominantly found in hematopoietic cells, this kinase is thought to be a novel target molecule for the treatment of GvHD. Adapted from “NFAT Signaling Pathway”, by Biorender.com (2021). Retrieved from https://app.biorender.com/biorender-templates.

TCR stimulation and its ligation, mediated through the contact of T-cells with APCs results in a dramatic increase of intracellular calcium (Ca^2+^) levels. Calcium influx is essential for activation, maturation and effector functions of T-cells. TCR engagement leads to the activation of PLC-γ, thereby increasing the intracellular levels of inositol 1,4,5-triphosphate (IP_3_). Binding of IP_3_ to its specific receptors in turn stimulates the release of calcium from intracellular storage compartments. Continuous depletion of intracellular calcium storage stimulates the opening of cell membrane based calcium channels to enhance the influx of calcium from the extracellular compartment. The intracellular increase of Ca^2+^ is required to activate calcium-dependent kinases and the transcription factor calcineurin, leading to activation of nuclear factor of activated T-cells (NFAT), thereby enhancing the transcription of a variety of different genes necessary for T-cell activation and effector functions (33, 149). However, a very strong increase of the intracellular cytoplasmic Ca^2+^ concentration leads to the transcription of pro-apoptotic signaling pathways and activation-induced cell death (AICD). The modulation of intracellular calcium levels was therefore hypothesized to be a potential therapeutic strategy for autoimmune diseases (149, 150). A major regulator of intracellular Ca^2+^ levels is the inositol 1,4,5-triphosphate 3-kinase (Itpk) family, comprising Itpka, Itpkb, Itpkc and inositol polyphosphate multikinase. The Itpk family acts as a negative regulator of Ca^2+^ influx through conversion of IP_3_ to inositol 1,3,4,5-tetrakisphosphate (IP_4_) and this regulatory mechanism is known to be highly important for T-cell development and survival (33–35, 151). Among all kinases in the Itpk family, Itpkb is most abundant in hematopoietic cells and genetic deficiencies of Itpkb lead to impaired T-cell development in the thymus, mainly based on AICD of immature CD4^+^CD8^+^ T-cells (34, 35, 152). Deletion of Itpkb in mature activated T-cells was shown to be a novel strategy to prevent T-cell driven autoimmunity through increase of intracellular calcium levels (**Figure 4**) (150). A recent study highlighted the therapeutic potential of Itpkb deletion and inhibition to control acute and chronic GvHD (149).

**Acute GvHD:** In major mismatch allo-HCT models, mice receiving allogeneic Itpkb-deleted T-cells survived significantly longer and experienced less weight loss compared to mice receiving T-cells with functional Itpkb. Complementary, histopathological analysis on day 7 after transplantation showed lower aGvHD pathology scores if mice received T-cells deficient for Itpkb compared to mice receiving wildtype T-cells. Consistent with these findings using a genetic approach, inhibition of Itpkb with GNF362 (150) prolonged the survival of mice receiving high doses of allogeneic T-cells (149). Itpkb deficiency caused reduced donor T-cell survival but no reduced inflammatory cytokine production (153). Itpkb^-/-^ T-cells had comparable or even higher intracellular tumor necrosis factor α (TNFα) and interferon γ (IFNγ) levels compared to Itpkb wildtype T-cells. However, quantitative T-cell analysis in the spleen, mesenteric lymph nodes and the small intestine and liver revealed lower numbers of Itpkb deficient T-cells compared to the WT control, whereas the proliferative capacity remained unchanged. Reduced numbers of donor Itpkb^-/-^ T-cells could be linked to lower T-cell survival, indicated by higher abundance of active caspase 8 in CD4^+^ and CD8^+^ T-cells from Itpkb^-/-^ donors after HCT (149, 154, 155). Since the survival of Itpkb deficient T-cells is impaired, GvL activity had to be investigated. Interestingly, mice receiving A20 leukemia cells and Itpkb^-/-^ T-cells survived significantly longer compared to mice receiving leukemia cells only or leukemia in combination with WT T-cells. Leukemia expansion was only seen in mice transplanted with leukemia cells only. The results indicate that Itpkb deficiency maintains anti-leukemia immunity while reducing aGvHD severity. Comparable results were achieved using the Itpkb inhibitor GNF362 (149).

**Chronic GvHD:** In comparison to T-cell mediated aGvHD, cGvHD establishes as an autoimmune-like disease (8, 11). Mice receiving Itpkb deficient T-cells had significantly improved pulmonary resistance, elastance and compliance compared to mice receiving WT T-cells. Comparable results were seen for lung and liver collagen, as well as for cGvHD histopathology scores. Mice with established cGvHD were also treated with the Itpkb inhibitor GNF362 and the investigators found improved pulmonary function, most probably based on reduced lung macrophage infiltration. Using a Scl-cGvHD model, Itpkb inhibition did result in reduced skin and liver histopathology scores, lower infiltration of proinflammatory macrophages and a reduction of IFNγ-producing T-cells.

In summary, the study could highlight the importance of Itpkb in regulating acute and chronic GvHD and delivered promising data indicating that pharmacological inhibition of Itpkb could be a potential novel therapeutic approach to control GvHD without impairing anti-leukemia immunity (149).

## TGFβ-Activated Kinase 1 (TAK1)

Dysregulated innate immune cells are key players in GvHD pathogenesis by activating APCs in the pro-inflammatory milieu (16) and inhibition of inflammatory cytokine signaling could ameliorate GvHD severity in mice and humans (15). Aiming to further understand the role and contribution of innate immune cells in GvHD, Kobayashi et al. performed gene expression profiling on monocytes from patients who experienced GvHD after allo-HCT (156). The investigators found increased expression of TGFβ-activated kinase 1 (TAK1) and downstream signaling molecules, including TNFα, IL-6 and IL-1β, in monocytes from patients with GvHD compared to patients who did not experience GvHD after allo-HCT. TAK1 is a member of the mitogen-activated kinase (MAPK) family and is a key regulating factor upstream of nuclear factor κB (NF-κB), c-Jun N-terminal kinase (JNK), extracellular signal-regulated kinase (ERK) and p38 in toll-like receptor (TLR) signaling (16). Based on its role in mediating inflammatory signaling, inhibition of TAK1 was hypothesized being a novel strategy to ameliorate GvHD severity by reducing pro-inflammatory signaling and T-cell alloreactivity (156).

**Acute GvHD:** Patient-derived monocytes were LPS-activated and treated with the TAK1 inhibitor 5Z-7-oxozeaenol (OZ), followed by analysis of cytokine production. Pro-inflammatory cytokine secretion was suppressed in a concentration-dependent manner. Application of OZ in a major mismatch murine aGvHD model prolonged the survival of GvHD mice, reduced GvHD scores and did result in lower serum levels of pro-inflammatory cytokines (156). In a different study, Mathew and Vinnakota et al. investigated the role of microglia in central nervous system (CNS) aGvHD and also found TAK1 being an important mediator of aGvHD-induced neurotoxicity (157). It could be shown for the first time that CNS-GvHD in mice is connected to an activation and expansion of microglia. Comparable, the numbers of microglia were higher in the grey and white matter of patients suffering from GvHD after allo-HCT compared to non-GvHD and non-HCT controls. Moreover, costimulatory molecules were increased on microglia from mice after allo-HCT compared to syngeneic (syn-) HCT and untreated controls. CNS-GvHD was connected to enhanced microglia-derived TNF production in mice and humans, driven by TAK1. Also, other inflammatory cytokines connected to TAK1 signaling were seen elevated in microglia after allo-HCT. Genetic depletion of TAK1 in microglia did alleviate CNS-GvHD associated pathology, as well as memory and cognitive deficits in mice after allo-HCT. Comparable results were seen using the TAK1 inhibitors Takinib and OZ. Takinib did even reduce IFNγ and IL-17 production of T-cells infiltrated into the brain. Based on these two studies and the finding that TAK1 inhibition does not interfere with anti-leukemia immunity after allo-HCT (157), TAK1 could be a novel and promising target to ameliorate aGvHD and CNS-GvHD (157).

## Mitogen-Activated Protein Kinase (MEK) Inhibition

The onset and pathogenesis of GvHD is correlated with strong TCR activation and stimulation of TCR downstream signaling pathways to enhance alloreactivity and cytokine production (158). Signaling through the rat sarcoma/mitogen-activated protein kinase kinase/extracellular-signal regulated kinase (RAS/MEK/ERK) cascade is also crucial to translocate transcription factors and to enable target gene transcription, cell proliferation, migration, survival and differentiation (**Figure 3**) (28, 159).

**Acute GvHD:** One pre-clinical study investigated activated signaling molecules in alloreactive T-cells isolated from mice suffering from aGvHD and identified significantly increased phosphorylation of ERK1/2 and STAT3 (160). Inhibition of ERK1/2 and STAT3 phosphorylation was thought to be a novel method to reduce donor T-cell alloreactivity (160). ERK1/2 phosphorylation was inhibited using the selective MEK1/2 inhibitor SL327. MEK1/2 is located upstream of ERK1/2 (161–163). SL327 could dose-dependently reduce the proliferation of T-cells upon CD3/CD28 stimulation and in a mixed lymphocyte reaction (MLR) (160, 164). A second preclinical study applied flow cytometry-based pERK1/2 analysis of human T-cells activated with PMA and Ionomycin and a preferential increase of ERK1/2 phosphorylation in naïve and central memory T-cells was seen (165). Application of U0126, a classical MEK inhibitor, and selumetinib, a second-generation MEK inhibitor, reduced ERK1/2 phosphorylation dose-dependently. The latter inhibitor is tested in various clinical trials for different cancer entities and was found safe to use with little to no hematologic toxicity (166–169). Besides proliferation, MEK inhibition reduced effector cytokine production by memory T-cells. Based on these results, the investigators aimed to elucidate the effect of MEK inhibition in an alloreactive setting. The proliferation of T-cells activated with allogeneic HLA-mismatched DCs was significantly suppressed by MEK inhibition while virus-specific T-cell responses were not affected (165). Comparable results were seen using the MEK inhibitor trametinib (170). Selumetinib suppressed cell division even stronger than the calcineurin inhibitor tacrolimus (165, 171). MEK inhibition was further evaluated in experimental major-mismatch aGvHD mouse models, where selumetinib significantly prolonged the survival of GvHD mice (165). The MEK inhibitor trametinib could also suppress GvHD in a xenogeneic model and enhanced the engraftment of diverse T-cell clones. In this model, MEK inhibition suppressed T-cell activity responsible for GvHD while promoting human T-cell reconstitution (172).

**Chronic GvHD:** In an additional preclinical analysis, trametinib treatment enhanced survival and reduced GvHD scores in a MHC-haploidentical GvHD model (173). MEK inhibition suppressed CD8^+^ T-cells and elevated naïve T-cells after allo-HCT. Trametinib also reduced target organ damage and lymphocyte infiltration. A second model confirmed the potency of MEK inhibition, as the development of cutaneous GvHD, skin sclerosis and alopecia was reduced upon trametinib application. Although trametinib was reported to be well-tolerated without toxicities *in vivo* (174), donor cell engraftment and myeloid immune reconstitution were suppressed. Since MEK inhibition was shown to suppress T-cell effector functions and proliferation, it is of high importance to investigate the effect on anti-tumor immunity. Surprisingly, MEK inhibition did not affect T-cell mediated anti-tumor immunity against mastocytoma cells, as T-cell transplanted mice survived longer compared to vehicle. Mice receiving tumor only without T-cells did not benefit from MEK inhibition, implicating that trametinib does not directly affect tumor cells. In comparison, the GvHD prophylaxis treatment tacrolimus shortened the survival of leukemia-bearing mice as it suppressed both, GvHD and GvL (173). Although preclinical results for MEK/ERK inhibition are promising in aGvHD and GvL models and MEK inhibitors were not reported having limiting toxicities in mice, to the best of our knowledge no clinical trial was initiated yet to evaluate the efficacy of MEK inhibition GvHD patients.

## AMP Kinase (AMPK)

GvHD following allo-HCT is predominantly driven by alloreactive donor T-cells which cause severe tissue damage (4). Novel therapeutic options aim to impair T-cell functions to reduce life-threatening GvHD without affecting GvL activity, including the idea to metabolically re-program T-cells after allo-HCT (175). Following transplantation, T-cells increase oxidative phosphorylation and fatty acid oxidation (176, 177). Since it is known that allogeneic effector T-cells require fatty acid oxidation (FAO) during GvHD, it was hypothesized that AMP kinase (AMPK) activation plays a major role in regulating T-cell activity after allo-HCT (175, 177).

**Acute GvHD:** Monlish and colleagues recently identified that alloreactive donor T-cells selectively increase AMPK activation during aGvHD after allo-HCT as they found elevated phosphorylation of AMPKα and the downstream molecule ACC in CD4^+^ and CD8^+^ T-cells isolated from mice on day 7 after allo-HCT (175). The investigators established AMPK double knockout (AMPK-dKO) mice lacking AMPKα1 and AMPKα2 in all peripheral T-cells. AMPK is a heterotrimeric molecule with the α subunit as kinase domain and the β/γ subunits being important for stability and substrate specificity (175, 178–180). Although T-cell development and *in vitro* proliferation in a MLR was not different between AMPK-dKO and WT T-cells, AMPK-deficient T-cells caused less severe GvHD after transplantation into lethally irradiated recipient mice. In two different GvHD models, the survival was prolonged and clinical GvHD scores were lower if mice received AMPK-dKO T-cells compared to WT T-cells (175). Comparable findings were described in a second study (181). In addition, the infiltration of AMPK-dKO T-cells into GvHD target organs was reduced. The anti-leukemia response was not impaired upon depletion of AMPK. Mechanistically, reduced GvHD was connected to reduced recovery and decreased expansion of AMPK-depleted T-cells after allo-HCT (175, 181). The fewer recovery was linked with increased apoptosis in mainly CD8^+^ T-cells, whereas the results are highly variable and the interpretation is therefore questionable. Although AMPK is a metabolic enzyme, depletion did not affect any investigated metabolic pathway, but rather affected other cell populations. Co-transplantation experiments revealed that AMPK-dKO T-cells stimulated an increase of WT Tregs. Given the importance of Tregs to reduce GvHD severity, increased Treg numbers due to accompanying AMPK-dKO cells were named as a major mechanism to suppress GvHD severity (175, 182–184). Although there are no AMPK inhibitors available yet, inhibition of AMPK in T-cells could serve as novel target for GvHD treatment; however, more detailed analysis is needed to better understand the role of AMPK after allo-HCT (175).

## p38 Mitogen-Activated Protein Kinase (MAPK)

The p38 mitogen-activated kinase (MAPK) is a major control mechanism for cellular responses, proliferation and cytokine production (17, 185, 186). Different p38 MAPK isoforms are expressed in most tissues and cell types and are activated by extracellular stimulatory signals, including inflammatory cytokines, growth signals and stress signals (17, 185, 187, 188). Activation of the p38 MAPK signaling cascade results target gene expression, including inflammatory mediators and cytokines (17, 188–190). Of all isoforms, p38α was reported as major regulator in inflammatory responses and therapeutic blockade with the p38α-specific inhibitor VX-702 was applied in rheumatoid arthritis (RA) to reduce inflammatory signals (191).

**Chronic GvHD:** Since enhanced p38 MAPK phosphorylation was found in fibroblasts from systemic sclerosis patients and p38 MAPK blockade reduced elevated type I collagen expression, the signaling pathway was hypothesized playing a role in sclerosis pathogenesis (17, 192, 193). Systemic sclerosis has various clinical similarities with Scl-cGvHD and therapeutic blockade of p38 MAPK signaling was thought to reduce cGvHD severity (17, 116, 194–196). Phosphorylation of p38 MAPK (Thr180/Tyr182) was increased in the skin of Scl-cGvHD mice compared to syngeneic BMT mice (17). Application of VX-702 delayed the onset of skin fibrosis and alopecia development and improved skin GvHD scores. The dermal thickness, collagen area and levels of procollagen I αI were reduced upon VX-702 treatment. More detailed analysis by histology and flow cytometry revealed reduced infiltration of CD4^+^ and CD8^+^ T-cells, as well as lower numbers of myeloid cells and macrophages into the skin of Scl-cGvHD mice upon p38 MAPK inhibition. Of all analyzed cytokines, only IL-6 and IL-13 were significantly reduced upon treatment, whereas major drivers of Scl-cGvHD, like TGFβ and IFNγ, were unchanged (17). However, complete tissue transcription profile analysis might mask some minor changes in immune cells and it would have therefore been better to analyze effector and inflammatory cytokine production on single cells levels and to screen for cytokines in the serum. Nevertheless, consistent with histology data, tissue RNA analysis revealed decreased expression of COL1A2 and fibronectin 1 upon p38 MAPK inhibition (17). In summary, the study showed that p38 MAPK is activated in cGvHD and therapeutic blockade could be a novel therapeutic intervention.

**Acute GvHD:** Although p38 MAPK inhibition seems promising in cGvHD, the treatment is questionable since reduced p38α MAPK levels (heterozygous p38α-KO) in donor grafts were found to accelerate acute intestinal GvHD in mice (197). Surprisingly, and contrary to the previously described cGvHD study, loss of donor p38 reinforced GvHD severity and reduced the survival of the mice. Cytokine analysis confirmed higher TNFα levels in the gut in allo-p38α^+/-^ grafted mice compared to the WT setting. Although p38α loss prolonged the survival of donor-derived intestinal intraepithelial lymphocytes *in vitro* and *in vivo*, donor lymphocyte expansion was decreased in the mesenteric lymph nodes upon p38α deficiency. Although the role of p38α-loss in the recipient compartment was not investigated, the study revealed a dichotomous effect of p38α in regulating inflammatory responses, cytokine expression, lymphocyte proliferation and intestinal GvHD after allo-HCT (197). Taken together with the before-mentioned study, the role of p38 MAPK in GvHD is still unclear and needs more detailed investigation, also comparing effects in chronic and acute GvHD as these could always be different (17, 197). Differences could be due to the models as the study on cGvHD applied an inhibitor, whereas the aGvHD study investigated the role of p38 MAPK using a genetic approach with p38-deficient donor cells.

## Aurora Kinase A

**Acute GvHD:** TCR activation and co-stimulation *via* CD28 stimulates mTOR and aurora kinase family signaling in T-cells, thereby activating substrates needed for T-cell proliferation (198). Transcriptomic analysis found increased expression of aurora kinase A in aGvHD patients and mice after allo-HCT. Inhibition of aurora kinase A prolonged survival and reduced GvHD scores in mice, however the animals could not be fully rescued (199). It was hypothesized that inhibition of aurora kinase A and JAK2 could be combined, as JAK2 activation by inflammatory stimuli leads to STAT3 activation and effector cytokine production (200). Betts et al. treated human MLRs with the JAK2 inhibitor TG101348, aurora kinase A inhibitor alisertib or a combination. T-cell proliferation was synergistically suppressed by the combination. Compounds targeting both kinases, had similar effects. Both kinase inhibitors reduced CD4^+^ and CD8^+^ T-cell activation *in vitro*, whereas the combination had the strongest impact. Also, T-cells produced less IL-17 and IFNγ upon kinase blockade. Interestingly, the induction of Tregs was significantly reduced upon kinase inhibition, but Tregs had potent inhibitory functions, mainly based on upregulation of surface CD39. Dual kinase inhibition caused higher ATP consumption, confirming the functionality of CD39 upregulation. Based on *in vitro* findings, the investigators applied JAK2 and aurora kinase A inhibitors in a xenogeneic aGvHD model. The survival of the recipients was significantly increased upon combination treatment, accompanied by lower GvHD scores. A novel dual inhibitor showed even stronger effects without impairing GvL activity. Based on these findings, more research is needed to further elucidate the role of aurora kinases in GvHD and their potency as novel targetable molecule (103).

## Conclusions and Outlook

In summary, the presented pre-clinical and clinical investigations reveal that kinase inhibition offers a huge variety of novel approaches to target both, acute and chronic GvHD. Since GvHD involves a vast number of pathways and signaling cascades for immune cell activation, proliferation and effector cytokine production, as well as in inflammatory signaling and fibrosis, the disease is hard to treat with only a single compound. Moreover, acute and chronic GvHD are completely different diseases and involve distinct pathways, making the treatment even more complicated. However, the involvement of different pathways is also a chance, presenting a variety of kinases as potentially targetable candidates. The great number of studies indicates how intensively researched kinases, the major signal transducers in immune cell signaling, are in the context of GvHD after allo-HCT. Although some compounds are already far in clinical trials, many questions remain unanswered, making deeper research necessary to unravel the potential of kinase inhibition in GvHD. Preclinical and clinical analyses revealed that treatment with single compounds has therapeutic limitations, as GvHD is mediated by a variety of pathways and not only by a single activated molecule. Novel therapeutic strategies should involve the combination of kinase inhibitors with other therapeutic interventions, as it is already investigated for JAK1 and JAK2 inhibitors with ECP. Moreover, different kinase inhibitors could be combined to potentiate the efficacy of the individual kinase inhibitors to enhance treatment success. JAK1 and/or JAK2 inhibitors, leading to reduced inflammatory cytokine production and decreased APC activation, could be combined with ROCK2 inhibition which potently blocks fibrosis and TFH formation in cGvHD. Also, ROCK2 blockade results in higher Treg numbers. Combinations of Syk, PI3K/mTOR and ITK/BTK could be beneficial as these kinases mediate early B- and T-cell activation. It might also be possible to apply kinase inhibitors sequentially to first hit a target being activated in aGvHD and target a second kinase to reduce the risk of aGvHD transforming into chronic GvHD. However, these strategies are still speculative and combination therapies should be carefully tested in preclinical models. Since many kinases are not only involved in disease but also in physiological signaling processes, the application and combination of inhibitors has the risk of potential side effects. Kinases such as MEK and MAPK are active in most cells and tissues and severe side effects are likely upon inhibition. Treatment-related adverse events have to be considered and highly specific molecules need to be designed to reduce off-target effects. So far, some kinases are only investigated pre-clinically, but should be tested clinically if effects are seen. After dose-finding studies, the inhibitors might first be investigated in SR-GvHD. Based on performance, the inhibitors might also be evaluated as first-line or prophylaxis therapies for patients after allo-HCT. However, transformation of these compounds into clinical trials is speculative as they first need to be critically evaluated in pre-clinical model. Taken together, the approval and clinical application of some kinase inhibitors, including the JAK1/2 inhibitor Ruxolitinib and the ROCK2 inhibitor belumosudil, is promising to better control acute and chronic GvHD after allo-HCT, thereby making allo-HCT available for more patients with severe hematological malignancies.

## Author Contributions

LMB and RZ developed the overall concept of this article. LMB collected and reviewed literature, discussed the studies and wrote the manuscript. RZ helped to write the manuscript and critically revised the manuscript. All authors contributed to the article and approved the submitted version.

## Funding

This article was supported by the Deutsche Forschungsgemeinschaft (DFG, German Research Foundation) – SFB-1479 – Project ID: 441891347, SFB TRR167, SFB850 (to RZ), by the Germany’s Excellence Strategy (CIBSS – EXC-2189 – Project ID 390939984 to RZ) and by ERC Consolidator grant (681012 GvHDCure to RZ). The article processing charge was funded by the Baden-Wuerttemberg Ministry of Science, Research and Art and the University of Freiburg in the funding program Open Access Publishing.

## Conflict of Interest

RZ received honoraria from Novartis, Incyte and Mallinckrodt.

The remaining author declares that the research was conducted in the absence of any commercial or financial relationships that could be construed as a potential conflict of interest.

## Publisher’s Note

All claims expressed in this article are solely those of the authors and do not necessarily represent those of their affiliated organizations, or those of the publisher, the editors and the reviewers. Any product that may be evaluated in this article, or claim that may be made by its manufacturer, is not guaranteed or endorsed by the publisher.
